# Molecular and Histopathological Study on the Ameliorative Impacts of *Petroselinum Crispum* and *Apium Graveolens* against Experimental Hyperuricemia

**DOI:** 10.1038/s41598-020-66205-4

**Published:** 2020-06-11

**Authors:** Mohamed Mohamed Soliman, Mohamed Abdo Nassan, Adil Aldhahrani, Fayez Althobaiti, Wafaa Abdou Mohamed

**Affiliations:** 1 Clinical Laboratory Sciences Department, Turabah University College, Taif University, Turabah, 29541 Saudi Arabia; 2 Biochemistry Department, Faculty of Veterinary Medicine, Benha University, Benha, 13736 Egypt; 3 Pathology Department, Faculty of Veterinary Medicine, Zagazig University, Zagazig, Egypt; 4 Department of Biotechnology, Faculty of Science, Taif University, Taif, Saudi Arabia; 5 Clinical Pathology Department, Faculty of Veterinary Medicine, Zagazig University, Zagazig, Egypt

**Keywords:** Biochemistry, Medical research

## Abstract

Hyperuricemia is an abnormal metabolic condition characterized by an increase in uric acid levels in the blood. It is the cause of gout, manifested by inflammatory arthritis, pain and disability. This study examined the possible ameliorative impacts of parsley (PAR) and celery (CEL) as hypouricemic agents at biochemical, molecular and cellular levels. PAR and CEL alone or in combination were orally administered to hyperuricemic (HU) mice and control mice for 10 consecutive days. Serum levels of uric acid and blood urea nitrogen (BUN), xanthine oxidase activity, antioxidants, inflammatory (IL-1β and TNF-α) and anti-inflammatory cytokines (IL-10) were measured. mRNA expression of urate transporters and uric acid excretion genes in renal tissues were examined using qRT-PCR (quantitative real time PCR). Normal histology and immunoreactivity of transforming growth factor-beta 1 (TGF-β1) in kidneys was examined. Administration of PAR and CEL significantly reduced serum BUN and uric acids in HU mice, ameliorated changes in malondialdehyde, catalase, and reduced glutathione, glutathione peroxidase (GPX), IL-1β, TNF-α and IL-10 in hyperuricemic mice. Both effectively normalized the alterations in mURAT-1, mGLUT-9, mOAT-1 and mOAT-3 expression, as well as changes in TGF-β1 immunoreactivity. Interestingly, combined administration of PAR and CEL mitigated all examined measurements synergistically, and improved renal dysfunction in the hyperuricemic mice. The study concluded that PAR and CEL can potentially reduce damaging cellular, molecular and biochemical effects of hyperuricemia both individually and in combination.

## Introduction

Hyperuricemia (HU) is defined as an increase in the levels of uric acid over normal ranges (6 mg/dL in females and 7 mg/dL in males)^1,2^. HU is associated with meat and seafood ingestion, hypertension and obesity^2–4^. Advanced HU is associated with gout^5^. Gout results in deposition of urate in soft tissues and joints, and arthritis in men over 40 years old^5^. Uric acid (UA) is the end product of the catabolism of purine compounds in the liver. UA is excreted mainly by the kidneys and to a lesser extent by the gastrointestinal tract^6,7^. It is degraded by gut microbiota (one third) in a process known as intestinal uricolysis^8^. The remaining two thirds depends on interchange between UA secretion and reabsorption in the kidney tubules^8–10^. Treatment of gout mainly depends on allopurinol (ALP). ALP is an inhibitor of xanthine oxidase and stimulates renal excretion of UA^10,11^. Other anti-inflammatory drugs (indomethacin) can be used, but these may cause side effects^11^. Therefore, identifying safe herbal medications is the goal for both patients and physicians.

The use of *organic* drugs and therapies is cost-effective^12^. The positive and promising effects of medicinal herbs on renal diseases, infertility, liver disorders and diabetes are clearly established and are accepted by patients and clinicians as a safe medication for these disorders^13–17^. Plants of medical importance contain flavonoids and other phenolic compounds that have strong antioxidant effects, and have been investigated in many studies^15–17^. Medicinal plants have fewer side effects compared to manufactured drugs and are often used as alternative medicine to counter the side effects of synthetic therapies^18,19^.

Parsley (*Petroselinum crispum, PAR*) is used as a spice, garnish or fragrance for cuisine across the world. Parsley is the most well-known antioxidant-rich herb that reduces inflammation, especially in the joints^20^. The leafy part is rich in polyphenols and has high antioxidant activity^21,22^. Parsley prevents cancer cells from differentiation and growth^23^, and is a safe, natural remedy to reduce glucose levels in diabetic individuals^24^.

Celery (*Apium graveolens L, CEL*) is known in the Middle East as Karafs. Celery seeds, leaves, and essential oil extracts are widely used in medicine. Phytochemical compounds extracted from celery include carbohydrates and phenols such as flavonoids, alkaloids and steroids^19^. Limonene, flavonoids, selinene, and frocoumarin glycosides are the main compounds present in celery, making it one of the most commonly used plants in traditional medicine^25^. Celery can prevent cardiovascular diseases, jaundice, liver diseases, and rheumatoid associated diseases^26^. Research on rats has shown that ethanolic extracts of celery leaves increases spermatogenesis^27^, improves fertility^28^ and has antifungal and anti-inflammatory properties^29^. Moreover, its seeds have therapeutic use in the treatment of bronchitis, fever, chronic skin disorders, and tumors^26–29^. Recent reports have confirmed celery’s lowering effect on UA levels^30^. However, the exact descriptive mechanism of such effects is still unclear. Therefore, this study aimed to investigate the anti-hyperuricemic activity of parsley and celery at cellular and molecular levels on experimental hyperuricemia induced by oxonate in mice.

## Materials and Methods

### Chemicals and kits

ALP, ethidium bromide, PO and agarose were purchased from Sigma-Aldrich (St. Louis, MO, USA). Reverse transcriptase enzymes and 100 bp DNA ladder were from MBI (Fermentas, Thermo Fisher Scientific, USA). Qiazol and Oligo dT primers were from QIAGEN (Valencia, CA, USA). The kits for MDA, catalase, GSH, GPx were from Biodiagnostic Co. (Dokki, Giza, Egypt). The kits for blood urea nitrogen (BUN), uric acid, creatinine, GOT and GPT were from EGY-CHEM for lab technology (Badr City, Egypt). Serum creatinine was measured using ELISA kit from Mybiosource (Cat. No. MBS763433, San Diego, CA 92195-3308, USA). Mouse IL-1 beta (Catalog No. E-EL-M0037), mouse TNF-alpha (Catalog No. E-EL-M0049), IL-10 ELISA kit (Catalog No. E-EL-M0046) and XO kits (Catalog No. E-BC-K024) were from Elabscience Biotechnology Inc., Memorial Drive, Suite 216, Houston, Texas 77079, USA.

### Preparation of parsley aqueous extract

Parsley was purchased from the local market in the Taif area, Saudi Arabia. Its identity was confirmed by a botanist (Prof. Yassin Alsudani) at the College of Science, Taif University. The leaves were washed in desalinated water and dried at room temperature in the dark. Parsley powders were diluted with distilled water (1:1 w/v) then given to the mice once daily at a dose of 7 g/kg bw. The remaining ground parsley was stored at −20 °C^20,31^.

### Preparation of celery aqueous extract

Fresh celery was identified by Prof. Yassin Alsudani (College of Science, Taif University). The whole plant was purchased from local Taif markets, KSA and kept for dryness in the dark. It was then ground into a fine powder. Two hundred grams of the powder was soaked in 70% ethanol for 2 days at 40 °C with gentle shaking. After centrifugation at 7000 × g, at 20 °C for 10 min, the supernatant was filtered. The solvent was removed using a rotary evaporator (Rotavapor R-300/R-300 Pro, https://www.buchi.com/rotavapor-r-300). The residue yield was 16% (w/w) and this was kept at −20 °C until use^30^.

### Animals, experimental design and samples collection

Swiss male mice were bought from the College of Pharmacy, King Abdel-Aziz University, Jeddah, Saudi Arabia. The Ethical Committee Office of Turabah University College, Taif University, Saudi Arabia, approved all procedures and *in vivo* animal use for this study. 56 male mice (7/group), aged 10 weeks and weighing 30–35 g were used. Mice were handled manually for one week to overcome handling stress prior to the onset of experiments. The animals were maintained in a dark/light cycle with free access to food and water. Group 1 was used as a control group and given free access to food and water. Group 2 was a positive HU group, injected PO intraperitoneally (250 mg/kg bw, daily at 8:00 am). The PO dosage and timing were determined as stated previously^20^. Group 3 was administered PO with an oral dose of allopurinol (ALP; 5 mg/kg bw daily, one hour after PO administration) for 10 days^32^. Group 4 was administered parsley at 7 g/kg bw orally as stated previously^31^. Group 5 was administered celery at 500 mg/kg bw orally as stated^30^. Groups 6 and 7 were administered PO at 8.00am, followed by PAR for group 6 and CEL for group 7 one hour later (9:00 am) for 10 days. Group 8 was administered PO at 8:00 am, followed by a combination of PAR and CEL at 9:00 am for 10 consecutive days. To overcome diethyl ether inhalation side effects, mice were fasted overnight then anaesthetized over 2 minutes using diethyl ether-soaked cotton in a 50 ml Falcon tube. Quickly, blood samples were taken from the eyes and the mice were then decapitated to collect further samples. Blood serum was stored at −20 °C; renal and hepatic tissue samples were preserved in Qiazol in anticipation of RNA extraction and gene expression analysis; and further kidney tissue samples were separated out for histopathology analysis and stored in 10% buffered neutral formalin.

### Xanthine Oxidase activity

The kit used depends on the catalysis of hypoxanthine to form xanthine and superoxide anion free radicals. In the presence of chromogenic agent and electronic receptors, it will form a purplish-red substance that can be measured at the OD value of 530 nm. For liver tissues, homogenate in 1:9 normal saline was placed on ice, centrifuged for 10 minutes and the supernatant used for XO assay. The measurement unit for serum is U/l and for liver is U/g protein tissue. The protocol used for XO is a partially modified version of the method used by Haidari *et al*.^31^

### Biochemical assays

Serum levels of the biomarkers specific to liver and kidney (GPT, GOT, uric acid, creatinine, BUN), cytokines (IL-1β, TNF-α and IL-10) and antioxidants (MDA, GSH, GPX and catalase) were measured using specific calorimetric commercial kits, following the relevant instruction manuals.

### Gene expression and quantitative real time PCR (qRT-PCR)

Total RNA was extracted from the kidney and liver tissues. RNA integrity was confirmed as stated previously^33^. Total RNA was denatured in Bio-Rad T100 Thermal Cycle at 70 °C for 5 minutes and reverse transcribed^33^. For qRT-PCR analysis, specific primers (Table 1) were designed using real-time Taqman primer design tool (https://www.genscript.com/tools/real-time-pcr-taqman-primer-design-tool). Each PCR reaction consisted of 1.5 μl of 1 μg/μl cDNA, 10 μl SYBR Green PCR Master Mix (Quanti Tect SYBR Green PCR Kit, Qiagen), 1 μM of each forward and reverse primer for each gene, and nuclease-free water to a final volume of 20 μl. Reactions were run and analyzed on an Applied Bio-system 7500 Fast Real time PCR Detection system. qRT-PCR conditions were: first denatured at 95 °C for 10 minutes, followed by 40 cycles at 95 °C for 15 seconds (second denaturation), then 60 °C for 60 seconds (annealing and extension stage). Variations in gene expression and intensity of examined genes were calculated from the obtained cycle threshold (CT) values provided by the real-time PCR instrumentation using the comparative CT method to beta-actin as a reference.

**Table 1:** The primers used for quantitative real time PCR (qRT-PCR). The primers sequence have been designed using real-time PCR (TaqMan) Primer and Probes Design Tool.

Gene	Product size (bp)	Accession number	Direction	Sequence (5′-3′)
mOAT-1	183	NM_008766.3	Sense	GACAGGGTCTCATCCCTAGC
			Antisense	GTCCCTGACACACTGACTGA
mOAT-3	153	NM_001164635.1	Sense	TACAGTTGTCCGTGTCTGCT
			Antisense	CTTCCTCCTTCTTGCCGTTG
mURAT-1	145	NM_009203.3	Sense	GATAGGTTTGGGCGCAGAAG
			Antisense	TCATCATGACACCTGCCACT
mGlut-9	153	NM_001102415.1	Sense	TTCGGGTCCTTCCTTCCTCTA
			Antisense	GGACACAGTCACAGACCAGA
mGda	139	NM_010266.2	Sense	GGCTGGTGGCTACTCCTATT
			Antisense	GCTTCCTCCAAGAGTGGCTA
mPNP	140	L11290.1	Antisense	CGACTGGTGTTTGGATTGCT
			Sense	ACCACCAAAGTTTCCACACC
mβ-actin	143	Nm_007393.5	Sense	CCAGCCTTCCTTCTTGGGTA
			Antisense	CAATGCCTGGGTACATGGTG

### Histological and immunohistochemistry analyses of kidney tissue

Kidney slices were dehydrated, embedded in paraffin and sectioned at 4 µm. Slides were stained with hematoxylin and eosin (H&E). Morphological changes were examined using a microscope (Nikon Eclipse 80i, Japan) and images were captured with a digital camera (Canon, SX620 HS - 20 MP, Japan). For immunohistochemistry, paraffin-embedded renal sections were deparaffinized, rehydrated and immersed in 2% H_2_O_2_ for 15 minutes (to inhibit peroxidase activity). Sections were then washed in phosphate buffer saline. Bovine serum albumin (5%) was used to block nonspecific binding sites. TGF-β1 polyclonal antibody was added for kidney slides in a dilution of 1:350 overnight at 4 °C. Slides were then incubated with secondary antibody after washing in PBS, developed with 3,3-diaminobezidine tetrahydrochloride then counterstained with hematoxylin. The percentage of positive immunoreactive cells for TGF-β1 was shown as the ratio of positively stained cells to the total cell number in the same field.

### Statistical analysis

Data are expressed as means ± standard error for values collected from 7 mice per group. One-way ANOVA was used to analyze the data. The probability level P < 0.05 and the individual comparisons were obtained using Duncan’s multiple range tests for SPSS software version 12.5 for Windows (SPSS, IBM, Chicago, IL, USA). P < 0.05 was considered statistically significant.

### Ethical statement

All procedures used in this study were carried out based on the NIH Guide for the care and use of laboratory animals. All precautions were followed to minimize animal suffering throughout the experiments.

## Results

### Administration of parsley and celery ameliorated liver and kidney dysfunction in hyperuricemic mice

Administration of PO increased serum levels of GOT, GPT, uric acid and BUN in hyperuricemic (HU) mice compared to normal control mice. ALP, PAR and CEL treated HU mice exhibited decreased altered parameters (Table 2). Co-administration of PAR and CEL showed a greater ameliorative effect (P < 0.05) on normalization of assayed parameters (Table 2) compared with PAR or CEL alone in HU mice.

**Table 2:** Ameliorative effects of parsley and celery on serum kidney and liver biomarkers in oxonate induced hyperuricemia.

	Creatinine (mg/dl)	BUN (mg/dl)	Uric acid (U/l)	GPT (U/l)	GOT (U/l)
Control	0.67 ± 0.05	11.1 ± 1	4.5 ± 0.2	33.5 ± 1.3	30.1 ± 1.7
HU	1.6 ± 0.15^*^	26.3 ± 1.3^*^	15.7 ± 0.9^*^	49.9 ± 2.1^*^	52 ± 3.0^*^
HU + ALP	0.7 ± 0.05^#^	14.9 ± 0.8^#^	8.01 ± 0.4^#^	37.3 ± 0.9^#^	36.9 ± 1.3^#^
Parsley	0.55 ± 0.01	12.6 ± 1.6	5.8 ± 0.6	33.1 ± 0.5	34.9 ± 1.7
Celery	0.6 ± 0.02	12.9 ± 1.5	5.7 ± 0.3	33.4 ± 1.7	35.1 ± 1.1
HU + Parsley	0.6 ± 0.02^#^	14.5 ± 1.13^#^	7.8 ± 0.4^#^	38.8 ± 1.2^#^	37.2 ± 1.4^#^
HU + Celery	0.6 ± 0.03^#^	13.5 ± 1.8^#^	6.9 ± 0.3^#^	36.8 ± 1.2^#^	36.4 ± 1.3^#^
HU + Par + CEL	0.5 ± 0.07^$^	10.4 ± 0.9^$^	5.3 ± 0.2^$^	29.3 ± 0.89^$^	27.1 ± 1.01^$^

Values are means ± standard error (SEM) for 7 different mice per each treatment. Values are statistically significant at *P < 0.05 vs control; ^#^P < 0.05 vs hyperuricemic group and ^$^P < 0.05 vs either hyperuricemic group or both HU + PAR or HU + CEL. ALP: Allopurinol; PAR: parsley; CEL celery; HU: hyperuricemia.

### Administration of parsley and celery decreased serum and hepatic Xanthine Oxidase (XO) activity in hyperuricemic mice

As shown in Table 3, serum and liver activities of XO were increased in HU mice and were normalized significantly (P < 0.05) in the ALP, PAR and CEL administered group compared to oxonate administered mice. Co-administration of both PAR and CEL induced additive inhibition in XO activity compared to ALP treated HU mice.

**Table 3:** Ameliorative effects of parsley and celery on serum and liver xanthine oxidase activity in hyperuricemic mice.

	Serum XO (U/l)	Hepatic XO (U/g tissue protein)
Control	13.9 ± 1.1	17.3 ± 1.2
HU	55.7 ± 4^*^	67.2 ± 6.8^*^
HU + ALP	24.4 ± 2.5^#^	27.1 ± 1.7^#^
Parsley	14.9 ± 1.3	17.5 ± 1.9
Celery	14.4 ± 2.9	15.4 ± 1.1
HU + Parsley	23.1 ± 4.7^#^	25.4 ± 2.4^#^
HU + Celery	22.2 ± 4.8^#^	29 ± 4.3^#^
HU + Par + CEL	17.8 ± 3.2^$^	20.1 ± 1.9^$^

Values are means ± standard error (SEM) for 7 different mice per each treatment. Values are statistically significant at *P < 0.05 vs control; ^#^P < 0.05 vs hyperuricemic group and ^$^P < 0.05 vs either hyperuricemic group or both HU + PAR or HU + CEL. ALP: Allopurinol; PAR: parsley; CEL celery; HU: hyperuricemia; XO: xanthine oxidase.

### Administration of parsley and celery ameliorated disorders in cytokine levels in hyperuricemic mice

Table 4 shows the changes in serum levels of IL-1β and TNF-α inflammatory cytokines, and IL-10 anti-inflammatory cytokine. Hyperuricemia induced a state of inflammation and significantly increased IL-1β and TNF-α levels (P < 0.05), while decreasing serum levels of IL-10. PAR and CEL administration ameliorated these effects. Co-administration of PAR and CEL induced a greater (P < 0.05) inhibitory effect on IL-1β and TNF-α, and a stimulatory effect on secretion of IL-10 (Table 4).

**Table 4:** Ameliorative effects of parsley and celery on serum levels of IL-1β, TNF-α and IL-10 in hyperuricemic mice.

	IL-1β (pg/ml)	TNF-α (pg/ml)	IL-10 (pg/ml)
Control	15.6 ± 1.1	21.5 ± 1.2	46.3 ± 2.1
HU	35.6 ± 1.8^*^	59.9 ± 4.6^*^	29.1 ± 1.8^*^
HU + ALP	17.7 ± 1.6^#^	32.1 ± 1.8^#^	42.5 ± 1.67^#^
Parsley	14.4 ± 2.4	28.2 ± 2	49.8 ± 1.88
Celery	13.8 ± 2.3	22.6 ± 1.59	50.1 ± 3.1
HU + Parsley	18.2 ± 1.7^#^	36.4 ± 2.6^#^	43.7 ± 1.5^#^
HU + Celery	19.2 ± 2.36^#^	31.1 ± 3.1^#^	42.9 ± 1.69^#^
HU + Par + CEL	13.3 ± 2.1^$^	26.4 ± 2.1^$^	45.8 ± 1.23^$^

Values are means ± standard error (SEM) for 7 different mice per each treatment. Values are statistically significant at *P < 0.05 vs control; ^#^P < 0.05 vs hyperuricemic group and ^$^P < 0.05 vs either hyperuricemic group or both HU + PAR or HU + CEL. ALP: Allopurinol; PAR: parsley; CEL celery; HU: hyperuricemia.

### Antioxidant activities of parsley and celery against oxidative stress associated with hyperuricemia in mice

Hyperuricemia increased tissue degradation by increasing MDA levels in the HU group (Table 5). These increases in MDA were normalized by PAR and CEL treatment. Hyperuricemia decreased catalase, and GSH and GPX levels but these returned to nearly control levels after PAR and CEL administration (Table 5). Co-administration of PAR and CEL to HU mice induced an additive ameliorative effect on the changes induced in measured antioxidants (Table 5).

**Table 5:** Ameliorative effects of parsley and celery on serum antioxidants levels in oxonate induced hyperuricemia.

	MDA (nmol/ml)	GSH (nmol/l)	GPX (U/l)	Catalase (U/l)
Control	13.2 ± 0.7	2.6 ± 0.1	161 ± 4.6	264 ± 22
HU	38.7 ± 1.01^*^	1.2 ± 0.1^*^	110 ± 3/5^*^	167 ± 13^*^
HU + ALP	16.5 ± 1^#^	2 ± 0.2^#^	147 ± 5.6^#^	254 ± 8.9^#^
Parsley	12.1 ± 1.3	3.1 ± 0.2	202 ± 29	309 ± 21.6
Celery	11.4 ± 0.5	3.1 ± 0.2	216 ± 31	287 ± 10.1
HU + Parsley	18.1 ± 1.5^#^	2.89 ± 0.1^#^	149 ± 6.1^#^	235 ± 15.9^#^
HU + Celery	19.3 ± 1.4^#^	3.1 ± 0.1^#^	156 ± 5.1^#^	227 ± 8^#^
HU + Par + CEL	12.5 ± 0.5^$^	3.4 ± 0.2^$^	184 ± 6.5^$^	257 ± 7.8^$^

Values are means ± standard error (SEM) for 7 different mice per each treatment. Values are statistically significant at *P < 0.05 vs control; ^#^P < 0.05 vs hyperuricemic group and ^$^P < 0.05 vs either hyperuricemic group or both HU + PAR or HU + CEL. ALP: Allopurinol MDA: Malondialdehyde; GSH: reduced glutathione; GPX: glutathione peroxidase; PAR: parsley; CEL celery; HU: hyperuricemia;

### Impacts of PAR and CEL on mRNA expression of genes associated with renal hyperuricemia

**Figure 1:**
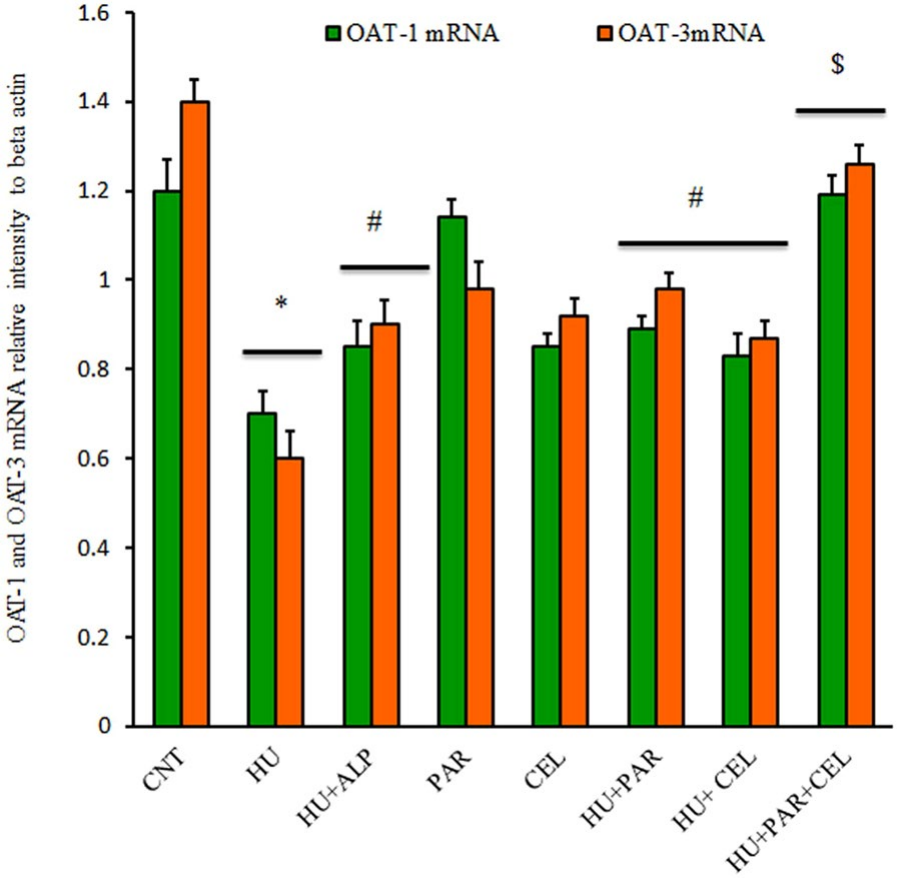
Ameliorative Effects of parsley and celery on changes of mRNA expression of OAT-1 and OAT-3 in HU mice using quantitative real time PCR. Graphic presentation of renal mRNA of OAT-1 and OAT-3 in different groups of mice after normalization with beta actin. *p < 0.05 vs control group; ^#^P < 0.05 vs HUR group and ^$^P < 0.05 vs either HU + Parsley or HU + Celery groups.

**Figure 2:**
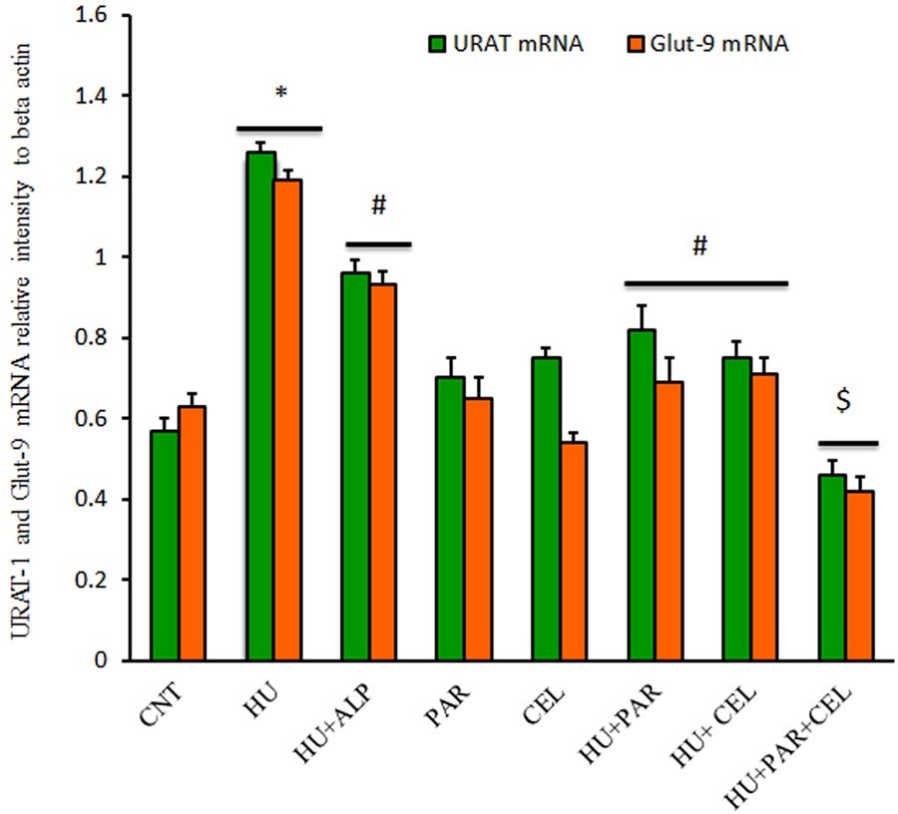
Ameliorative Effects of parsley and celery on mRNA expression of mURAT-1 and mGlut-9 in HU mice using real time PCR. Graphic presentation of renal mRNA of mURAT-1 and mGlut-9 in different groups of mice after normalization with beta actin. *p < 0.05 vs control group; ^#^P < 0.05 vs HUR group and ^$^P < 0.05 *vs* either HU + parsley or HU + celery groups.

We examined mRNA expression of mOAT-1, mOAT-3, mURTA-1 and mGlut9 genes responsible for urate excretion and reabsorption in the kidney. Figure 1 shows significant oxonate down-regulation in mRNA expression of mOAT-1 and mOAT-3, and significant (p < 0.05) up-regulation in mURAT-1 and mGlut-9 mRNA expression in HU mice kidneys compared with the control group. The alteration in mRNA expression of urate transporter-related genes was consistent with the elevation of serum uric acid and BUN levels reported in Table 2. PAR and CEL administration alone showed significant down-regulation in mURAT-1 and mGlut-9 and up-regulation in mOAT-1 and mOAT-3 mRNA expression (Figs. 1 and 2). The additive synergistic effect on altered genes was clear when both PAR and CEL were co-administered for the HU group.

### Impacts of PAR and CEL on mRNA expression of liver genes associated with uric acid metabolism

**Figure 3:**
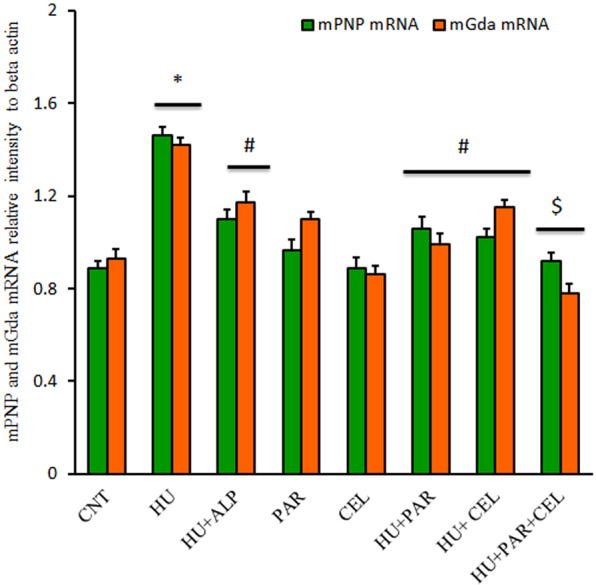
Ameliorative Effects of parsley and celery on mRNA expression of mPNP and mGda HU mice using real time PCR. Graphic presentation of liver mRNA of mPNP and mGda in different groups of mice after normalization with beta actin. *p < 0.05 vs control group; ^#^P < 0.05 vs HUR group and ^$^P < 0.05 vs either HU + parsley or HU + celery groups.

We examined mRNA expression of mice PNP and mice guanine Gda genes responsible for uric acid metabolism in the liver. As shown in Fig. 3, oxonate administration induced significant up-regulation in mPNP and mGda mRNA expression in HU mice (p < 0.05) compared to the control group. PAR and CEL regulated the alteration reported in HU groups. There was an additive synergistic effect for PAR and CEL when administered together to HU mice (Fig. 3).

### Impacts of parsley and celery on renal histology and TGF-β1 immunoreactivity in hyperuricemic mice

#### Kidney histology

**Figure 4:**
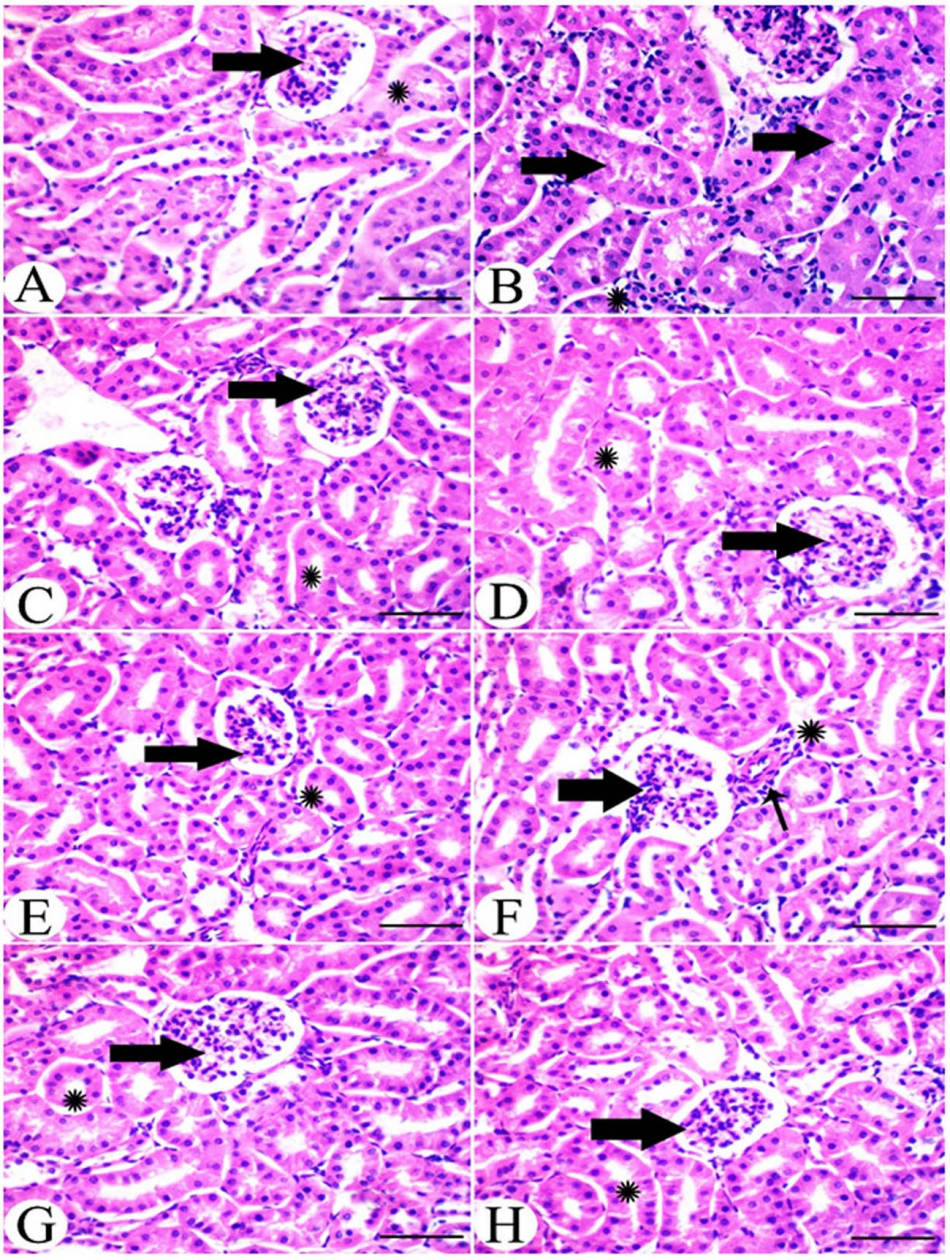
(**A**) Kidney of control group showing normal glomerular (arrow) and tubular (*) structure. (**B**) Kidney of HU group showed occlusion of tubular lumina by dense cell infiltration (arrow) and a shrinkage of glomerular tufts with periglomrular and interstitial (*) round cells infiltration. (**C**) Kidney of HU group treated with allopurinol showed normal glomerular architecture (arrow) with normal tubular histology (*). (**D**) Kidney of parsley administered mice showed normal renal tissue with normal tubular (arrow) and glomerular picture (*). Kidney of celery administered group showed the normal histological picture of both glomerular (arrow) and tubular (*) sections (**F**) Kidney of HU group treated with parsley alone showed restoration of normal picture with normal glomerular (thick arrow) and tubular (*) structure and mild perivascular round cells infiltration (thin arrow). (**G**) Kidney of HU group treated with celery showed restoration of glomerular (arrow) and tubular (*) tissue histology. H. Kidney of HU group treated with celery and parsley showed normal histological picture of both glomerular (arrow) and tubular (*) tissue with absence of urate crystals. Scale bar = 50 μm.

Control mice kidneys showed normal glomerular and tubular structure (Fig. 4A), whereas HU group kidneys showed a dense eosinophilic mass occluding the tubular lumina as well as leukocytic infiltration (Fig. 4B). Shrunken glomerular tufts, and periglomerular and interstitial (*) round cells infiltration were also observed. HU group kidneys treated with allopurinol showed normal glomerular architecture with normal tubular histology (Fig. 4C). Kidneys of parsley administered mice showed normal renal tissue with a normal tubular and glomerular picture (Fig. 4D). Kidneys of celery administered mice showed the normal histological picture of both glomerular and tubular sections (Fig. 4E). Kidneys of the HU group treated with parsley alone showed restoration of the normal picture with mild perivascular round cells infiltration (Fig. 4F). Kidneys of the HU group treated with celery showed restoration of glomerular and tubular tissue histology (Fig. 4G). Kidneys of the HU group treated with celery and parsley showed a normal histological picture of both glomerular and tubular tissue (Fig. 4H).

#### Immunoreactivity of renal TGF-β1

**Figure 5:**
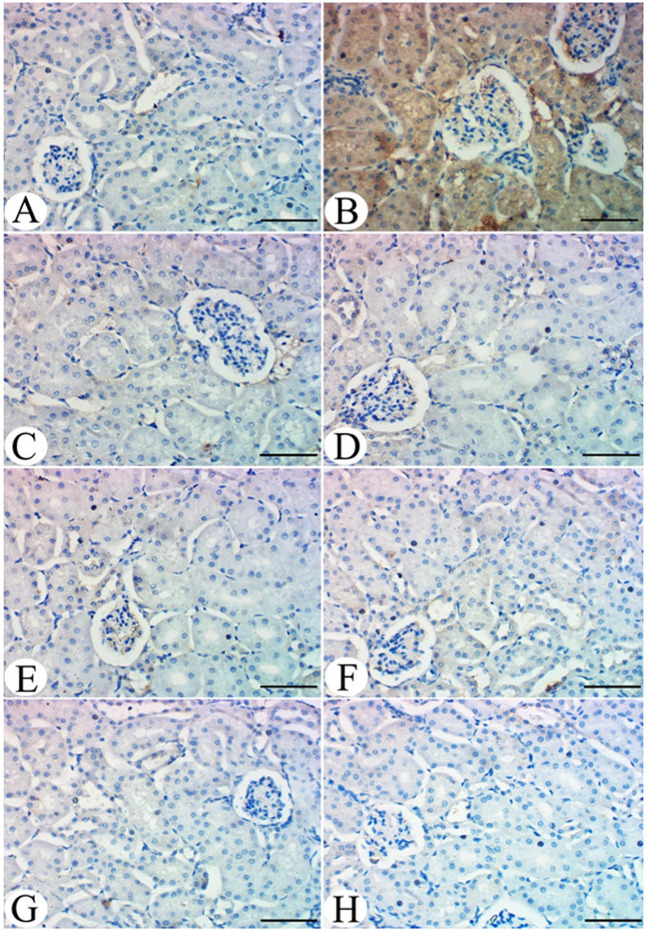
(**A**) Kidney of control group showed absence of expression of TGF-β1 in renal tissue. (**B**) Kidney of HU group showed increased expression of TGF-β1 in renal tubular tissue. (**C**) Kidney of HU group treated with allopurinol showed no marked expression of TGF-β in renal tissue. (**D**) Kidney of parsley administered mice showed absence of expression of TGF-β1 in renal tubular tissue. (**E**) Kidney of celery group showed absence of TGF-β1 expression in tubular tissue. (**F**) Kidney of HU group treated with parsley alone showed no observed reactivity for TGF-β1 in renal tissue. (**G**) Kidney of HU group treated with celery showed glomerular and tubular tissue with no TGF-β1 expression. H. Kidney of HU group treated with celery and parsley together showed restoration of normal picture without expression of TGF-β1 in renal tissue. Scale bar = 50 μm.

Kidneys of the control group showed an absence of TGF-β1 expression in renal tissue (Fig. 5A). Kidneys of the HU group showed high intensity and immunoreactivity for TGF-β1 in renal tubular tissue (Fig. 5B). Kidneys of the HU group treated with allopurinol showed no marked expression of TGF-β1 in renal tissue (Fig. 5C). Kidneys of parsley administered HU mice showed an absence of expression of TGF-β1 in renal tubular tissue (Fig. 5D). Kidneys of the celery group showed an absence of TGF-β1 expression in tubular tissue (Fig. 5E). Kidneys of the HU group treated with parsley alone showed no observed reactivity for TGF-β1 in renal tissue (Fig. 5F). Kidneys of the HU group treated with celery showed glomerular and tubular tissue with no TGF-β1 expression (Fig. 5G). Kidneys of the HU group treated with celery and parsley together showed more restoration in renal cells without expression of TGF-β1 (Fig. 5H). Table 6, shows particularly high intensity scores for TGF-β1 expression in the PAR and CEL administered HU groups.

**Table 6:** Immunohistochemical scoring and intensity of TGF-β in renal sections of different treated groups.

Immunohistochemical scoring of TGF-β	CNT	HU	HU + ALP	PAR	CEL	HU + PAR	HU + CEL	HU + PAR + CEL
	0	4	0	0	0	0	0	0
Staining intensity	−	+++	−	−	−	−	−	−

Score 1 = (No expression; no positive stained cells per examined three high-power fields (HPF), at 40× magnification.

Score 2 = (weak; 1–10 positive stained cells/HPF).

Score 3 = (moderate; 11–20 positive stained cells/HPF).

Score 4 = (strong; >20 positive stained cells/HPF).

## Discussion

The results suggested that parsley and celery are safe herbal remedies that can be used either alone or in combination^30,31^ to lower the effects on serum levels of uric acid and xanthine oxidase activity in hyperuricemic mice. Hyperuricemia increases the production of oxygen free radicals, induces lipid peroxidation, and up-regulates inflammatory and down-regulates anti-inflammatory cytokine expression and secretion^32,34,35^. Herbal plants can increase antioxidant content in experimental animals and rodents^24,36^. The major functions of most flavonoids present in medicinal plants are their ability to scavenge free radicals and increase antioxidant activities^37,38^. As shown in previous reports, medicinal plants increased total antioxidant capacity, suppressed reactive oxygen species (ROS) and prevented damage induced by oxidative stress^39,40^. Here, parsley and celery showed the potency to improve and increase antioxidant activities, eliminate tissue destruction and reduce inflammatory effects of hyperuricemia.

A negative correlation between the levels of antioxidants and XO activity has been confirmed in patients with acute herbicide poisoning^41^. XO is the key enzyme responsible for catalytic synthesis of uric acid from xanthine and hypoxanthine^42^, and is responsible for ROS generation^43^. Consequently, higher amounts of ROS are generated alongside uric acid production^43^.

Therefore, the suppressive effects of parsley and celery on experimental hyperuricemia may be attributed to inhibition of oxidative stress. Deposition of urate crystals in the kidney and joints stimulates inflamed cells to produce IL-1β, which promotes the release of a series of inflammatory cytokines (TNF-α and IL-6)^44^, causing a state of general inflammation^45^. Furthermore, patients with hyperuricemia exhibit decreased levels of the anti-inflammatory cytokine, IL-10^46^. Clinical trials have also shown that gout is associated with elevated IL-1β^47^. These alterations in cytokine levels were ameliorated by PAR and CEL administration either alone or in combination; the combination effect was more effective.

In this study, parsley and celery reduced inflammatory cytokines (IL-1β and TNFα), enhanced serum antioxidant activities and eliminated pathological changes in the kidney. The results suggest that the effect of parsley and celery on IL-1β and TNF-α may be through the modulation of oxidative stress and the enhancement of antioxidant activities. Celery contains furocoumarins, flavonoids (apigenin), phenolic compounds and tannins^48^. The hyperuricemic and xanthine oxidase inhibitory activity of celery was investigated to a lesser extent. Lin *et al*.^49^ reported *in vitro* studies that apigenin interacts with XO in its active site.

**Figure 6:**
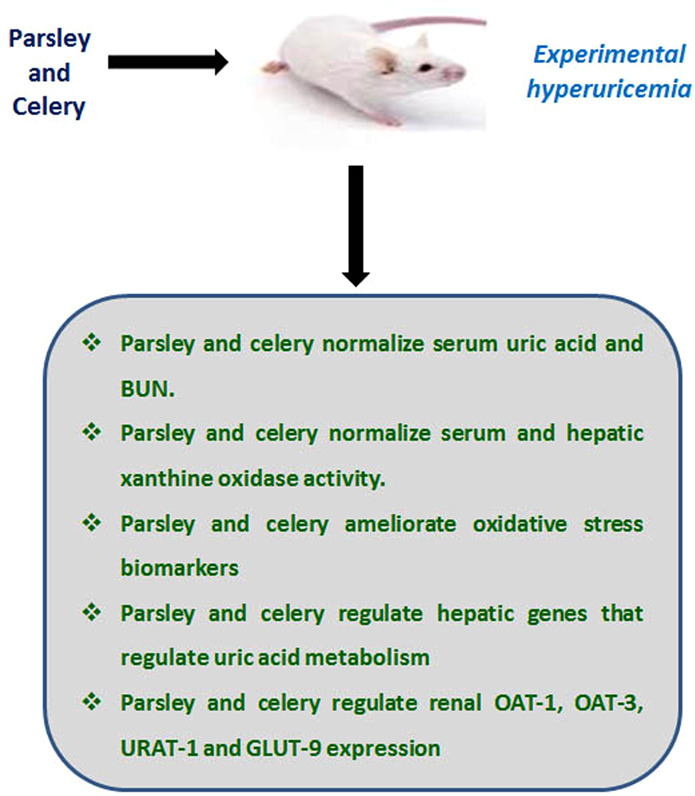
Schematic illustration for the ameliorative effects of parsley and celery on experimental hyperuricemia.

Several transporter genes play critical roles in urate secretion and excretion during hyperuricemia. URAT1, a renal urate anion exchanger and an integral membrane protein found primarily in kidney, transports urate across the proximal convoluted tubules^50,51^. Its expression depends on the uric acid levels in the blood. mGlut-9 is another urate transporter that regulates urate transport through the proximal tubules^52^. OAT-1 and OAT-3 are localized in the proximal convoluted tubules (in the basolateral membrane)^53^. OAT-1 plays a role in the uptake and secretion of urate^53^. OAT-3 participates in the cellular uptake of urate and in urate secretion. URAT1, OAT-1 and OAT-3 have recently been considered the ideal targets for hyperuricemia treatment^54^. This study is the first to show that parsley and celery have the potential to regulate urate excretion associated genes (URAT1, GLUT-9, OAT-1 and OAT-3), either alone or in combination. PO administration significantly up-regulated mURAT1 and mGlut-9 expression, and down-regulated mOAT-1 and mOAT-3 expressions in mouse kidneys. Oxonate-induced urate reabsorption and reduced urate secretion is counteracted by parsley and celery, which, when co-administered, reduced the effect of disorders associated with hyperuricemia. Both PAR and CEL effectively cured hyperuricemia through: control of xanthine oxidase activity, control of inflammatory cytokines, increase in antioxidant activities and decrease in oxidative stress. Further, the genes responsible for urate transporter expression were controlled. These effects are illustrated in Fig. 6.

## Conclusion

The effects of experimentally induced hyperuricemia were controlled and regulated through administering parsley and/or celery. These herbs are a safe and effective treatment and their effect is heightened when co-administered. Figure 6 shows the cellular, biochemical and molecular effects of the treatment.

## Supplementary information

Supplementary information.

Supplementary information.

## Data Availability

The data of the current study are available on reasonable request.
