# Supplementary material for: Molecular and Histopathological Study on the Ameliorative Impacts of Petroselinum Crispum and Apium Graveolens against Experimental Hyperuricemia
Source: Sci Rep. 2020 Jun 11;10:9512. doi: 10.1038/s41598-020-66205-4 (PMC7289838; doi:10.1038/s41598-020-66205-4)
Supplement: Supplementary file 1 — Supplementary information. [file 41598_2020_66205_MOESM1_ESM.docx]

**References**

1 Li, L. *et al.* Is hyperuricemia an independent risk factor for new-onset chronic kidney disease?: A systematic review and meta-analysis based on observational cohort studies. *BMC Nephrol* **15**, 122, doi:10.1186/1471-2369-15-122 (2014).

2 Lapi, F., Azoulay, L., Yin, H., Nessim, S. J. & Suissa, S. Concurrent use of diuretics, angiotensin converting enzyme inhibitors, and angiotensin receptor blockers with non-steroidal anti-inflammatory drugs and risk of acute kidney injury: nested case-control study. *Bmj* **346**, e8525, doi:10.1136/bmj.e8525 (2013).

3 Choi, H. K., Atkinson, K., Karlson, E. W., Willett, W. & Curhan, G. Purine-rich foods, dairy and protein intake, and the risk of gout in men. *N Engl J Med* **350**, 1093-1103, doi:10.1056/NEJMoa035700 (2004).

4 Zhu, J. N., Qi, X. Y., Tan, Y. & Lyu, X. H. [Dietary Factors Associated with Hyperuricemia and Glycolipid Metabolism Disorder in Middle-aged and Elderly People]. *Sichuan Da Xue Xue Bao Yi Xue Ban* **47**, 68-72 (2016).

5 Weaver, A. L. Epidemiology of gout. *Cleve Clin J Med* **75 Suppl 5**, S9-12, doi:10.3949/ccjm.75.suppl_5.s9 (2008).

6 de Oliveira, E. P. & Burini, R. C. High plasma uric acid concentration: causes and consequences. *Diabetol Metab Syndr* **4**, 12, doi:10.1186/1758-5996-4-12 (2012).

7 Rock, K. L., Kataoka, H. & Lai, J. J. Uric acid as a danger signal in gout and its comorbidities. *Nat Rev Rheumatol* **9**, 13-23, doi:10.1038/nrrheum.2012.143 (2013).

8 Maiuolo, J., Oppedisano, F., Gratteri, S., Muscoli, C. & Mollace, V. Regulation of uric acid metabolism and excretion. *Int J Cardiol* **213**, 8-14, doi:10.1016/j.ijcard.2015.08.109 (2016).

9 Bobulescu, I. A. & Moe, O. W. Renal transport of uric acid: evolving concepts and uncertainties. *Adv Chronic Kidney Dis* **19**, 358-371, doi:10.1053/j.ackd.2012.07.009 (2012).

10 Jalal, D. I., Chonchol, M., Chen, W. & Targher, G. Uric acid as a target of therapy in CKD. *Am J Kidney Dis* **61**, 134-146, doi:10.1053/j.ajkd.2012.07.021 (2013).

11 Terkeltaub, R. Update on gout: new therapeutic strategies and options. *Nat Rev Rheumatol* **6**, 30-38, doi:10.1038/nrrheum.2009.236 (2010).

12 Al-Asmari, A. K., Athar, M. T. & Kadasah, S. G. An Updated Phytopharmacological Review on Medicinal Plant of Arab Region: Apium graveolens Linn. *Pharmacogn Rev* **11**, 13-18, doi:10.4103/phrev.phrev_35_16 (2017).

13 Kooti, W. *et al.* The effect of hydro-alcoholic extract of celery on male rats in fertility control and sex ratio of rat offspring. **16**, 43-49 (2014).

14 Popovic, M., Kaurinovic, B., Trivic, S., Mimica-Dukic, N. & Bursac, M. Effect of celery (Apium graveolens) extracts on some biochemical parameters of oxidative stress in mice treated with carbon tetrachloride. *Phytother Res* **20**, 531-537, doi:10.1002/ptr.1871 (2006).

15 Lone, Z. A., Lone, Y., Khan, S. S., Wani, A. A. & Reshi, M. I. J. J. o. M. P. R. Hepatoprotective medicinal plants used by the Gond and Bhill tribals of District Raisen Madhya Pradesh, India. **9**, 400-406 (2015).

16 Wu, S. Y. *et al.* An emerging translational model to screen potential medicinal plants for nephrolithiasis, an independent risk factor for chronic kidney disease. *Evid Based Complement Alternat Med* **2014**, 972958, doi:10.1155/2014/972958 (2014).

17 Kooti, W., Farokhipour, M., Asadzadeh, Z., Ashtary-Larky, D. & Asadi-Samani, M. The role of medicinal plants in the treatment of diabetes: a systematic review. *Electron Physician* **8**, 1832-1842, doi:10.19082/1832 (2016).

18 Asadi-Samani, M., Kooti, W., Aslani, E. & Shirzad, H. A Systematic Review of Iran's Medicinal Plants With Anticancer Effects. *J Evid Based Complementary Altern Med* **21**, 143-153, doi:10.1177/2156587215600873 (2016).

19 Gauri, M., Ali, S. J. & Khan, M. S. J. I. A. I. M. A review of Apium graveolens (Karafs) with special reference to Unani medicine. **2**, 131-136 (2015).

20 Rahmat, A., Ahmad, N. S. S., Ramli, N. S. J. O. P. & Medicine, E. Parsley (Petroselinum crispum) supplementation attenuates serum uric acid level and improves liver and kidney structures in oxonate-induced hyperuricemic rats. 1-9 (2018).

21 Marin, I., Sayas-Barbera, E., Viuda-Martos, M., Navarro, C. & Sendra, E. Chemical Composition, Antioxidant and Antimicrobial Activity of Essential Oils from Organic Fennel, Parsley, and Lavender from Spain. *Foods* **5**, doi:10.3390/foods5010018 (2016).

22 Wong, P. Y. & Kitts, D. D. J. F. c. Studies on the dual antioxidant and antibacterial properties of parsley (Petroselinum crispum) and cilantro (Coriandrum sativum) extracts. **97**, 505-515 (2006).

23 Mafuvadze, B. *et al.* Apigenin prevents development of medroxyprogesterone acetate-accelerated 7,12-dimethylbenz(a)anthracene-induced mammary tumors in Sprague-Dawley rats. *Cancer Prev Res (Phila)* **4**, 1316-1324, doi:10.1158/1940-6207.Capr-10-0382 (2011).

24 Farzaei, M. H., Abbasabadi, Z., Ardekani, M. R., Rahimi, R. & Farzaei, F. Parsley: a review of ethnopharmacology, phytochemistry and biological activities. *J Tradit Chin Med* **33**, 815-826, doi:10.1016/s0254-6272(14)60018-2 (2013).

25 Kooti, W. *et al.* The effects of hydro-alcoholic extract of celery on lipid profile of rats fed a high fat diet. **8**, 325-330 (2014).

26 Sowbhagya, H., Srinivas, P. & Krishnamurthy, N. J. F. c. Effect of enzymes on extraction of volatiles from celery seeds. **120**, 230-234 (2010).

27 Kooti, W., Mansouri, E., Ghasemiboroon, M., Harizi, M. & Amirzargar, A. Protective effects of celery (Apium Graveolens) on testis and cauda epididymal spermatozoa in rat. *Iran J Reprod Med* **12**, 365-366 (2014).

28 Marzouni, H. Z., Daraei, N., Sharafi-Ahvazi, N., Kalani, N. & Kooti, W. J. W. J. P. P. S. The effects of aqueous extract of celery leaves (Apium graveolens) on fertility in female rats. **5**, 1710-1714 (2016).

29 Mencherini, T. *et al.* An extract of Apium graveolens var. dulce leaves: structure of the major constituent, apiin, and its anti-inflammatory properties. *The Journal of pharmacy and pharmacology* **59**, 891-897, doi:10.1211/jpp.59.6.0016 (2007).

30 Dolati, K. *et al.* Inhibitory Effects of Apium graveolens on Xanthine Oxidase Activity and Serum Uric Acid Levels in Hyperuricemic Mice. *Preventive nutrition and food science* **23**, 127-133, doi:10.3746/pnf.2018.23.2.127 (2018).

31 Haidari, F., Keshavarz, S. A., Mohammad Shahi, M., Mahboob, S. A. & Rashidi, M. R. Effects of Parsley (Petroselinum crispum) and its Flavonol Constituents, Kaempferol and Quercetin, on Serum Uric Acid Levels, Biomarkers of Oxidative Stress and Liver Xanthine Oxidoreductase Aactivity inOxonate-Induced Hyperuricemic Rats. *Iranian journal of pharmaceutical research : IJPR* **10**, 811-819 (2011).

32 Billiet, L., Doaty, S., Katz, J. D. & Velasquez, M. T. Review of hyperuricemia as new marker for metabolic syndrome. *ISRN Rheumatol* **2014**, 852954, doi:10.1155/2014/852954 (2014).

33 Saad, D. Y., Soliman, M. M., Baiomy, A. A., Yassin, M. H. & El-Sawy, H. B. Effects of Karela (Bitter Melon; Momordica charantia) on genes of lipids and carbohydrates metabolism in experimental hypercholesterolemia: biochemical, molecular and histopathological study. *BMC Complement Altern Med* **17**, 319, doi:10.1186/s12906-017-1833-x (2017).

34 Balakumar, P., Sharma, R., Kalia, A. & Singh, M. J. C. H. R. Hyperuricemia: is it a risk factor for vascular endothelial dysfunction and associated cardiovascular disorders? **5**, 1-6 (2009).

35 Kanellis, J. *et al.* Uric acid stimulates monocyte chemoattractant protein-1 production in vascular smooth muscle cells via mitogen-activated protein kinase and cyclooxygenase-2. **41**, 1287-1293 (2003).

36 Kolarovic, J., Popovic, M., Zlinska, J., Trivic, S. & Vojnovic, M. Antioxidant activities of celery and parsley juices in rats treated with doxorubicin. *Molecules* **15**, 6193-6204, doi:10.3390/molecules15096193 (2010).

37 Nickavar, B., Kamalinejad, M. & Izadpanah, H. In vitro free radical scavenging activity of five Salvia species. *Pak J Pharm Sci* **20**, 291-294 (2007).

38 Yao, Y., Sang, W., Zhou, M. & Ren, G. Phenolic composition and antioxidant activities of 11 celery cultivars. *J Food Sci* **75**, C9-13, doi:10.1111/j.1750-3841.2009.01392.x (2010).

39 Nguyen, M. T. *et al.* Xanthine oxidase inhibitory activity of Vietnamese medicinal plants. *Biol Pharm Bull* **27**, 1414-1421, doi:10.1248/bpb.27.1414 (2004).

40 Zummah, A. & Martha, R. D. J. M. O. T. Antihyperurisemic Activity of Aqueous Celery Infusion by Xanthine Oxidase Enzyme Inhibition. **23**, 131-136.

41 Zhang, J., Lv, G. & Zhao, Y. The significance of serum xanthine oxidase and oxidation markers in acute paraquat poisoning in humans. *Clin Biochem* **44**, 221-225, doi:10.1016/j.clinbiochem.2010.09.006 (2011).

42 Ramallo, I. A., Zacchino, S. A., Furlan, R. L. J. P. A. A. I. J. o. P. C. & Techniques, B. A rapid TLC autographic method for the detection of xanthine oxidase inhibitors and superoxide scavengers. **17**, 15-19 (2006).

43 Oguz, N., Kirca, M., Cetin, A. & Yesilkaya, A. Effect of uric acid on inflammatory COX-2 and ROS pathways in vascular smooth muscle cells. *J Recept Signal Transduct Res* **37**, 500-505, doi:10.1080/10799893.2017.1360350 (2017).

44 Sabina, E. P. & Rasool, M. An in vivo and in vitro potential of Indian ayurvedic herbal formulation Triphala on experimental gouty arthritis in mice. *Vascul Pharmacol* **48**, 14-20, doi:10.1016/j.vph.2007.11.001 (2008).

45 Jeong, J. H. *et al.* CD14(+) Cells with the Phenotype of Infiltrated Monocytes Consist of Distinct Populations Characterized by Anti-inflammatory as well as Pro-inflammatory Activity in Gouty Arthritis. *Front Immunol* **8**, 1260, doi:10.3389/fimmu.2017.01260 (2017).

46 Li, S. *et al.* Antigouty arthritis and antihyperuricemia properties of celery seed extracts in rodent models. *Molecular medicine reports* **20**, 4623-4633, doi:10.3892/mmr.2019.10708 (2019).

47 Zeng, M. *et al.* IL-37 inhibits the production of pro-inflammatory cytokines in MSU crystal-induced inflammatory response. *Clin Rheumatol* **35**, 2251-2258, doi:10.1007/s10067-015-3109-5 (2016).

48 Kooti, W., Daraei, N. J. J. o. e.-b. c. & medicine, a. A review of the antioxidant activity of celery (Apium graveolens L). **22**, 1029-1034 (2017).

49 Lin, C. M., Chen, C. S., Chen, C. T., Liang, Y. C. & Lin, J. K. Molecular modeling of flavonoids that inhibits xanthine oxidase. *Biochem Biophys Res Commun* **294**, 167-172, doi:10.1016/s0006-291x(02)00442-4 (2002).

50 Li, S. *et al.* Antigouty arthritis and antihyperuricemia properties of celery seed extracts in rodent models. *Mol Med Rep*, doi:10.3892/mmr.2019.10708 (2019).

51 Enomoto, A. *et al.* Molecular identification of a renal urate anion exchanger that regulates blood urate levels. *Nature* **417**, 447-452, doi:10.1038/nature742 (2002).

52 Vitart, V. *et al.* SLC2A9 is a newly identified urate transporter influencing serum urate concentration, urate excretion and gout. *Nat Genet* **40**, 437-442, doi:10.1038/ng.106 (2008).

53 Ichida, K. *et al.* Urate transport via human PAH transporter hOAT1 and its gene structure. *Kidney Int* **63**, 143-155, doi:10.1046/j.1523-1755.2003.00710.x (2003).

54 So, A. & Thorens, B. Uric acid transport and disease. *J Clin Invest* **120**, 1791-1799, doi:10.1172/jci42344 (2010).
