# Supplementary material for: Molecular and Histopathological Study on the Ameliorative Impacts of Petroselinum Crispum and Apium Graveolens against Experimental Hyperuricemia
Source: Sci Rep. 2020 Jun 11;10:9512. doi: 10.1038/s41598-020-66205-4 (PMC7289838; doi:10.1038/s41598-020-66205-4)
Supplement: Supplementary file 2 — Supplementary information. [file 41598_2020_66205_MOESM2_ESM.docx]

**Molecular and Histopathological Study on the Ameliorative Impacts of *Petroselinum Crispum* and *Apium Graveolens* against Experimental Hyperuricemia**

Mohamed Mohamed Soliman^1,2*^; Mohamed Abdo Nassan^3^; Adil Aldhahrani^1^; Fayez Althobaiti^4^; Wafaa Abdou Mohamed^5^

^1^ Clinical Laboratory Sciences Department, Turabah University College, Turabah 29541, Taif University, Saudi Arabia. adildh@hotmail.com; [mohamedsoliman8896@yahoo.com](mailto:mohamedsoliman8896@yahoo.com);

^2^ Biochemistry Department, Faculty of Veterinary Medicine, Benha University, Benha 13736, Egypt. [mohamed.soliman@fvtm.bu.edu.eg](mailto:mohamed.soliman@fvtm.bu.edu.eg)

^3^ Pathology Department, Faculty of Veterinary Medicine, Zagazig University, Zagazig, Egypt. [moh_nassan@yaho.com](mailto:moh_nassan@yaho.com)

^4^ Department of  Biotechnology, Faculty of Science, Taif University, Taif, Saudi Arabia. faizh1394@gmail.com

^5^ Clinical Pathology Department, Faculty of Veterinary Medicine, Zagazig University, Zagazig, Egypt. [waffa.clinical@yahoo.com](mailto:waffa.clinical@yahoo.com)

Correspondence to: Mohamed Mohamed Soliman; Biochemistry Department, Faculty of Veterinary Medicine, Benha University, Benha 13736, Egypt.

Email: [mohamed.soliman@fvtm.bu.edu.eg](mailto:mohamed.soliman@fvtm.bu.edu.eg)

Tel: 00201223722404; 00966501531640

`
